# Data for the qualitative modeling of the osmotic stress response to NaCl in *Escherichia coli*

**DOI:** 10.1016/j.dib.2016.09.028

**Published:** 2016-09-22

**Authors:** Delphine Ropers, Aline Métris

**Affiliations:** a Inria, Grenoble – Rhône-Alpes Research Center, Saint Ismier, France; b Institute of Food Research, Norwich Research Park, Norwich NR4 7UA, UK

**Keywords:** Systems biology, Osmotic stress, Dynamic modelling, Piecewise-linear approximations

## Abstract

Qualitative modeling approaches allow to provide a coarse-grained description of the functioning of cellular networks when experimental data are scarce and heterogeneous. We translate the primary literature data on the response of *Escherichia coli* to hyperosmotic stress caused by NaCl addition into a piecewise linear (PL) model. We provide a data file of the qualitative model, which can be used for simulation of changes of protein concentrations and of DNA coiling during the physiological response of the bacterium to the stress. The qualitative model predictions are directly comparable to the available experimental data. This data is related to the research article entitled “Piecewise linear approximations to model the dynamics of adaptation to osmotic stress by food-borne pathogens” (Metris et al., 2016) [1].

**Specifications Table**

**Table:** 

Subject area	*Computational biology*
More specific subject area	*Qualitative modeling of the dynamics of a gene regulatory network*
Type of data	*Graph, figures, table, model equations, model files (GNAML and SBML formats)*
How data was acquired	*Simulations were performed by means of the publicly available tool Genetic Network Analyzer*
Data format	*Equations, GNAML and SBML files*
Experimental factors	*Primary literature data*
Experimental features	*Gene expression and transcription factor binding sites of E. coli and Salmonella during osmotic stress response*
Data source location	*Inria, Saint Ismier, France*
Data accessibility	*The data is with this article*

**Value of the data**

• The reconstruction of the osmotic stress response network of *E. coli* provides a compilation of current knowledge on this process.
• The piecewise-linear model of the network is useful to exploit the heterogeneous and scarce experimental data on hyper-osmotic stress: their comparison with the model predictions allow to verify if we have a good understanding of the network functioning or if additional hypotheses should be formulated to reconcile potential discrepancies.
• The model can be easily extended to describe the response of *E. coli* to alternative osmotic stresses, caused for instance by other humectants and low moisture.

## 1 Data

The data provided in this article include a reconstruction of the hyper-osmotic stress response network of *Escherichia coli* and its translation into a piece-wise linear model. Files with the model equations in tow formats (GNAML and SBML) are also given, for computer simulation of the physiological response of *E. coli* to the presence or the absence of salt.

## 2 Experimental design, materials and methods

**Fig. 1:**
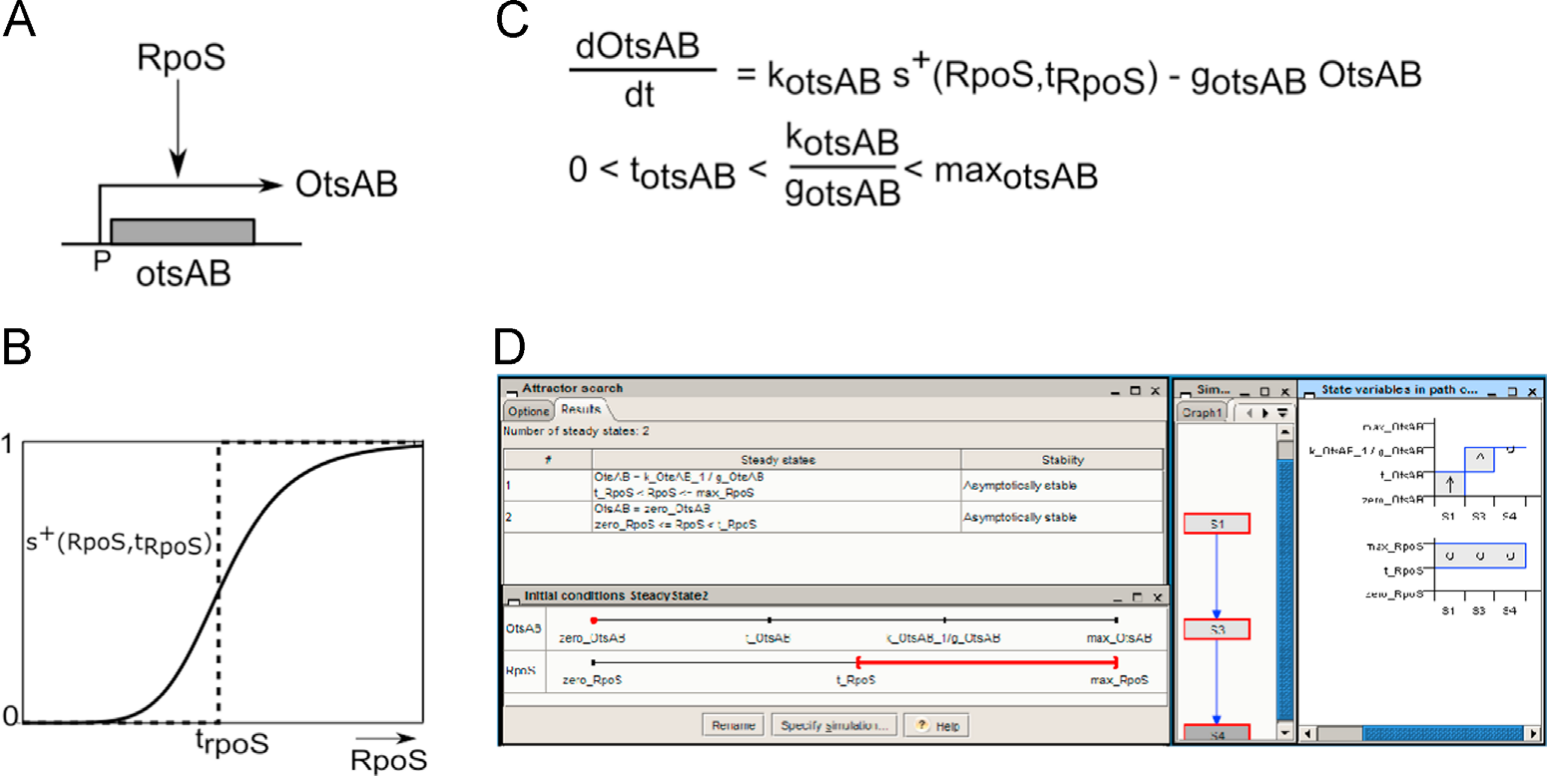
PL modeling of a simple network. (A) Network reconstruction from literature data. The expression of the operon genes *otsA* and *otsB* is RpoS-dependent [3]. Trehalose synthesis is considered to be under the control of OtsAB, the product of a single gene *otsAB*, whose promoter P is recognized by RpoS. (B) Positive step function (dashed line) used to approximate the sigmoidal Hill function (continuous line) often found in the regulation of gene expression. (C) PL model of the simple network and ordering of the threshold and focal parameters. (D) Screenshot of the initial conditions for simulation and of the state transition graph, attractor search and simulation results returned by Genetic Network Analyzer.

The PL modeling of the osmotic stress response network is briefly described below (see Batt et al. [2] and references therein for more information) and it is illustrated with a simple example in Fig. 1. Four steps were necessary before we could generate predictions on the network behavior that could be compared to experimental data.

### 2.1 Reconstruction of the osmotic stress response network

Our analysis of the physiological response of *E. coli* to hyper-osmotic stress is centered around proteins and markers known to play a key role in this process. Based on an extensive search of the literature and previous work [4], [5], we have reconstructed a network of eight genes: the sigma factor RpoS, the transcription factors Fis, IHF, and CRP, the symporter ProP, the trehalose synthase OtsAB, and markers of the osmotic stress response, OsmY, and of cellular growth, the stable RNAs. The assumptions made to reconstruct the network and the role of the different network components and their interactions are summarized in Table 1. The reader is referred to Metris et al. [1] for additional information. An example is provided in Fig. 1A, in the case of the genes involved in the synthesis of the osmoprotectant trehalose, *otsA* and *otsB*. Expression of these genes is both osmotically and growth-phase regulated in a RpoS-dependent manner as determined by gene expression of mutants in Hengge-Aronis et al. [3]. Since the two genes are organized in an operon and share the same regulation [6], we consider OtsAB to be the product of a single gene *otsAB*, whose promoter P is recognized by RpoS.

### 2.2 Translation of the gene regulatory network into a PL model

We consider the network as composed of four different modules, each one accomplishing a specific task, that of setting (1) the concentration of the potassium salt of glutamate and the DNA supercoiling level; (2) the concentration of the general stress response factor RpoS; (3) the concentration of the complex CRP–cAMP; (4) the concentration of OsmY and the growth rate. We do not model explicitly the concentration of RNA polymerase nor the concentration of the σ^70^ factor, as they are not known to vary in response to osmotic stress. We indicate in the table below the model equations and corresponding parameter ordering. The notations will follow the convention used above, *k*, representing protein synthesis rates, *g*, degradation rates, and *t,* threshold parameters.

Regulatory influences are described by means of step functions that change value at a threshold concentration. These functions simplify the sigmoidal Hill functions generally used to describe cooperative processes involved in the regulation of gene expression. For instance, the positive influence of RpoS on *otsAB* expression is described by a positive step function (see Panel B of Fig. 1). It evaluates to 1 when RpoS is above its threshold concentration t_RpoS_, and to 0 otherwise. Negative step functions are used to describe cooperative inhibition of gene expression. Hence, the auto-inhibition of IHF expression is described by the step function $s^{-}\left(\mathrm{IHF},t_{\mathrm{ihf}}^{2}\right)$, which equals to 1 when IHF is below its threshold value $t_{\mathrm{ihf}}^{2}$ and to 0 otherwise (see Table 1).

PL equations describe the rate of change of protein or RNA concentrations as the difference between their synthesis rate and their degradation rate. For instance, the PL equation for OtsAB in Panel C of Fig. 1 states that OtsAB is synthesized at a rate k_otsAB_ when RpoS is above its threshold concentration, while the synthesis rate is null in the absence of RpoS. OtsAB is degraded at a basal rate g_otsAB_ OtsAB. The concentration to which OtsAB tends when it is synthesized, $\frac{k_{\mathrm{otsAB}}}{g_{\mathrm{otsAB}}}$, should be above its threshold level: $\frac{k_{\mathrm{otsAB}}}{g_{\mathrm{otsAB}}}>t_{\mathrm{otsAB}}$; otherwise the protein would never reach a level above which it is able to produce trehalose and, indirectly, to affect the efflux of potassium.

We represent the input signal, i.e. the application of an osmotic stress to the system, by a step function $s^{+}\left(u,t_{u}\right)$ which equates to 1 upon osmotic shock. It captures the molecular changes induced by osmotic stress such as supercoiling and accumulation of glutamate as explained in Section 2.1 in Metris et al. [1].

### 2.3 Qualitative simulation of the PL model

The model in Table 1 has been implemented in and simulated with the publicly available software tool Genetic Network Analyzer (GNA 8.4, Genostar, http://userclub.genostar.com/en/genostar-software/gnasim.html). The corresponding model file is given in the supplementary data in GNAML and qualitative SBML formats [20]. An example of qualitative simulation with the simple network model is given in Panel D of Fig. 1.

Running the attractor search functionality of GNA shows that there are two stable steady states for the osmotic stress response model, one characteristic for normal growth in the absence of osmotic stress and one reached in case of osmotic stress. We can simulate how the system responds to salt addition and leaves the non-stressed state for the stressed one, by perturbing the state of normal growth with the signal of stress switched on $\left(u>t_{u}\right)$. The simulation returns a state transition graph composed of 192 states, showing all the possible dynamic behaviors of the system going from the initial state of normal growth to the stressed state.

### 2.4 Comparison of model predictions with experimental data

Each path in the state transition graph describes the evolution of protein and RNA concentrations, which can be confronted to the experimental data [1]. For instance, literature data summarized in Table 2 suggest an accumulation of OtsA and OtsB as trehalose accumulates after a hyper-osmotic stress induced by salt [21].

The expression varies with the conditions and the strains, however the pattern remains similar; a delay before increased expression during adaptation. The trend is also similar for *E. coli* and *Salmonella typhimurium* , so, in this case, the same equation may be used for both species. In the simple example of Fig. 1, the concentration of protein OtsAB is predicted to increase once RpoS accumulates in the cell. This prediction is hence consistent with experimental data. This type of analysis may be carried out for the different osmotic genes with more complex regulation as shown in Metris et al. [1].

## Figures and Tables

**Table 1:** Equations of the PL model with corresponding assumptions and parameter ordering.

Module	State variable	Assumptions	Equation	Ordering
Potassium glutamate/DNA supercoiling module	ProP, transporter of proline and glycine betaine	2 promoters [7], the σ^70^ dependent promoter P1, inhibited by the complex CRP–cAMP [8] and the σ^S^-dependent promoter P2 stimulated by proteins Fis and CRP–cAMP [9], [10]	$\frac{d}{\mathrm{dt}}\mathrm{ProP}=k_{\mathrm{proP}}^{1}\left(1-s^{+}\left(\mathrm{CRP},t_{\mathrm{crp}}^{3}\right)s^{+}\left(u,t_{u}\right)\right)+k_{\mathrm{ProP}}^{2}s^{+}\left(\mathrm{CRP},t_{\mathrm{crp}}^{3}\right)s^{+}\left(u,t_{u}\right)\;\times s^{+}\left(\mathrm{Fis},t_{\mathrm{fis}}^{3}\right)s^{+}\left(\mathrm{RpoS},t_{\mathrm{rpoS}}\right)-g_{\mathrm{proP}}\mathrm{ProP}$	$0<t_{\mathrm{proP}}<\frac{k_{\mathrm{proP}}^{1}}{g_{\mathrm{proP}}}<\frac{k_{\mathrm{proP}}^{2}}{g_{\mathrm{proP}}}<\max_{\mathrm{proP}}$
			$k_{\mathrm{proP}}^{1}$ and $k_{\mathrm{proP}}^{2}$, synthesis rates for each promoter	
	OtsAB, trehalose synthase	σ^S^-dependent [3]	$\frac{d}{\mathrm{dt}}\mathrm{OtsAB}=k_{\mathrm{otsAB}}s^{+}\left(\mathrm{RpoS},t_{\mathrm{rpoS}}\right)-g_{\mathrm{otsAB}}\mathrm{OtsAB}$	$0<t_{\mathrm{otsAB}}<\frac{k_{\mathrm{otsAB}}}{g_{\mathrm{otsAB}}}<\max_{\mathrm{otsAB}}$
RpoS module	RpoS, general stress factor	intracellular concentration assumed to come from cellular stabilization (see [1] for additional information)	$\frac{d}{\mathrm{dt}}\mathrm{RpoS}=k_{\mathrm{rpo}S}^{1}-\left(g_{\mathrm{rpoS}}^{1}+g_{\mathrm{rpoS}}^{2}s^{-}\left(u,t_{u}\right)\right)\mathrm{RpoS}$	$0<\frac{k_{\mathrm{rpoS}}}{g_{\mathrm{rpoS}}^{1}+g_{\mathrm{rpoS}}^{2}}<t_{\mathrm{rpoS}}<\frac{k_{\mathrm{rpoS}}}{g_{\mathrm{rpoS}}^{1}}<\max_{\mathrm{rpoS}}$
			$g_{\mathrm{rpoS}}^{1}$ and $g_{\mathrm{rpoS}}^{2}$, degradation rate constants, one unregulated, and one modulated by osmotic stress	
CRP–cAMP module	CRP, global regulator	Active in transcription when bound to the cAMP [11]. Transcription of P1 is inhibited by Fis [12]	$\frac{d}{\mathrm{dt}}\mathrm{CRP}=k_{\mathrm{crp}}^{1}s^{+}\left(\mathrm{CRP},t_{\mathrm{crp}}^{1}\right)s^{+}\left(u,t_{u}\right)s^{-}\left(\mathrm{Fis},t_{\mathrm{fis}}^{2}\right)+k_{\mathrm{crp}}^{2}-g_{\mathrm{crp}}\mathrm{CRP}$	$0<t_{\mathrm{crp}}^{1}<t_{\mathrm{crp}}^{2}<\frac{k_{\mathrm{crp}}^{2}}{g_{\mathrm{crp}}}<t_{\mathrm{crp}}^{3}<\frac{k_{\mathrm{crp}}^{1}+k_{\mathrm{crp}}^{2}}{g_{\mathrm{crp}}}\;<\max_{\mathrm{crp}}$
			$k_{\mathrm{crp}}^{1}$ and $k_{\mathrm{crp}}^{2}$, synthesis rates for each promoter	
Output module	*rrn*, stable RNAs	P1 is stimulated by Fis. Both promoters are assumed to be repressed by osmoprotectants [1], [13], [14].	$\frac{d}{\mathrm{dt}}\mathrm{rrn}=k_{\mathrm{rrn}}^{1}s^{+}\left(\mathrm{Fis},t_{\mathrm{fis}}^{1}\right)\left(1-s^{+}\left(u,t_{u}\right)s^{-}\left(\mathrm{OtsAB},t_{\mathrm{otsAB}}\right)s^{-}\left(\mathrm{ProP},t_{\mathrm{proP}}\right)\right)\;+k_{\mathrm{rrn}}^{2}\left(1-s^{+}\left(u,t_{u}\right)s^{-}\left(\mathrm{OtsAB},t_{\mathrm{otsAB}}\right)s^{-}\left(\mathrm{ProP},t_{\mathrm{proP}}\right)\right)-g_{\mathrm{rrn}}\mathrm{rrn}$	$0<\frac{k_{\mathrm{rrn}}^{2}}{g_{\mathrm{rrn}}}<\frac{k_{\mathrm{rrn}}^{1}+k_{\mathrm{rrn}}^{2}}{g_{\mathrm{rrn}}}<\max_{\mathrm{rrn}}$
			$k_{\mathrm{rrn}}^{1}$ and $k_{\mathrm{rrn}}^{2}$, synthesis rates for each promoter	
	Fis, factor for inversion stimulation	Expression is stimulated by IHF and repressed by both CRP–cAMP and Fis itself [1], [15], [16].	$\frac{d}{\mathrm{dt}}\mathrm{Fis}=k_{\mathrm{fis}}s^{+}\left(\mathrm{IHF},t_{\mathrm{ihf}}^{1}\right)\left(1-s^{+}\left(\mathrm{CRP},t_{\mathrm{crp}}^{2}\right)s^{+}\left(u,t_{u}\right)\right)s^{-}\left(\mathrm{Fis},t_{\mathrm{fis}}^{4}\right)-g_{\mathrm{fis}}\mathrm{Fis}$	$0<t_{\mathrm{fis}}^{1}<t_{\mathrm{fis}}^{2}<t_{\mathrm{fis}}^{3}<t_{\mathrm{fis}}^{4}<\frac{k_{\mathrm{fis}}}{g_{\mathrm{fis}}}<\max_{\mathrm{fis}}$
	IHF, integration host factor	σ^S^-dependent and inhibits itself	$\frac{d}{\mathrm{dt}}\mathrm{IHF}=k_{\mathrm{ihf}}^{1}+k_{\mathrm{ihf}}^{2}s^{+}\left(\mathrm{RpoS},t_{\mathrm{rpoS}}\right)s^{-}\left(\mathrm{IHF},t_{\mathrm{ihf}}^{2}\right)-g_{\mathrm{ihf}}\mathrm{IHF}$	$0<t_{\mathrm{ihf}}^{1}<\frac{k_{\mathrm{ihf}}^{1}}{g_{\mathrm{ihf}}}<t_{\mathrm{ihf}}^{2}<\frac{k_{\mathrm{ihf}}^{1}+k_{\mathrm{ihf}}^{2}}{g_{\mathrm{ihf}}}<\max_{\mathrm{ihf}}$
			$k_{\mathrm{ihf}}^{1}$and $k_{\mathrm{ihf}}^{2}$correspond to IHF synthesis mediated by σ^70^ and σ^S^ respectively	
	OsmY, osmotically induced membrane protein	σ^S^ responsible for *osmY* expression under osmotic stress [17], [18], [19]	$\frac{d}{\mathrm{dt}}\mathrm{OsmY}=k_{\mathrm{osmY}}^{1}{s^{+}\left(u,t_{u}\right)s^{-}\left(\mathrm{OtsAB},t_{\mathrm{otsAB}}\right)s^{-}\left(\mathrm{ProP},t_{\mathrm{proP}}\right)+k_{\mathrm{osmY}}^{2}s}^{+}\left(\mathrm{RpoS},t_{\mathrm{rpoS}}\right)s^{+}\left(u,t_{u}\right)\times s^{-}\left(\mathrm{OtsAB},t_{\mathrm{otsAB}}\right)s^{-}\left(\mathrm{ProP},t_{\mathrm{proP}}\right)\;-g_{\mathrm{osmY}}\mathrm{OsmY}$	$0<\frac{k_{\mathrm{osmY}}^{1}}{g_{\mathrm{osmY}}}<\frac{k_{\mathrm{osmY}}^{1}+k_{\mathrm{osmY}}^{2}}{g_{\mathrm{osmY}}}<\max_{\mathrm{osmY}}$
			$k_{\mathrm{osmY}}^{1}$and $k_{\mathrm{osmY}}^{2}$correspond to OsmY synthesis mediated by σ^70^ and σ^S^ respectively	

**Table 2:** Literature survey of the dynamic response of *otsAB* operon following a hyper-osmotic stress.

Protein (reference)	Conditions	Method	Strains	Time					
OtsA [22]	LB with 5% NaCl compared to LB with no salt	Decimal logarithm of the gene transcript relative to 16S gene measured by qRT-PCR		15 min.			60 min.		
			*E. coli* K303	<0.3 (log(2))			<1.5		
			*E. coli* K356	>−1			<1		
			*E. coli* K331-4	<0.3 (log(2))			<1		
			*E. coli* N09-1298	>−1			>1		
			*E. coli* FAM21843	>0.3 (log(2))			>1		

OtsB [23]	glucose minimal medium with 0.4 M NaCl in aerobic conditions	Ratio of protein induction measured by two dimensional gel electrophoresis	*E. coli* MG1655	0 min	10 min		30 min		60 min
				0	0		>0		4.5

OtsA [24]	glucose minimal medium with 0.4 M NaCl in aerobic conditions		*E. coli* MG1655	9 min					
		Ratio of gene expression as measured by micro-array as compared to no added NaCl in the medium		1.5					
		Ratio obtained by Northern blot analysis		8.1					
	LB with 6% NaCl	Fold change in gene expression as measured by micro-array as compared to no added NaCl in the medium	*S. Typhimurium* 4/74	0 h		6 h		24 h	
			
OtsA [25]				<3		6.6		<3	
OtsB [25]				<3		8.1		<3	

OtsB [26]	defined medium+0.3 M NaCl	mRNA level normalized to 16S as measured by qRT-PCR	*S. Typhimurium* LT2	0 min		20 min		60 min	
				0		>1		3	
