# Supplementary material for: Data for the qualitative modeling of the osmotic stress response to NaCl in Escherichia coli
Source: Data Brief. 2016 Sep 22;9:606–12. doi: 10.1016/j.dib.2016.09.028 (PMC5066198; doi:10.1016/j.dib.2016.09.028)
Supplement: Supplementary file 1 — Supplementary material [file mmc1.pdf]

Grenoble, 18th August 2015

The authors declare that they have no competing interests. The manuscript has been read and approved by all named authors.

Dr. Delphine Ropers (corresponding author)

Email: delphine.ropers@inria.fr

Dr. Aline Métris

Email: aline.metris@ifr.ac.uk

RESEARCH CENTRE
GRENOBLE - RHÔNE-ALPES
Inovallée
655 avenue de l'Europe
CS 90051
38334 Montbonnot Cedex
Phone: +33 (0)4 76 61 52 00
Fax: +33 (0)4 76 61 52 52

www.inria.fr
